# Lp Quasi-norm Minimization: Algorithm and Applications

**DOI:** 10.21203/rs.3.rs-3632062/v1

**Published:** 2023-11-28

**Authors:** Omar M. Sleem, M.E. Ashour, N. S. Aybat, Constantino M. Lagoa

**Affiliations:** 1 Department of Electrical Engineering, Pennsylvania State University, State College, PA, 16802 USA.; 2 Wireless R&D Department, Qualcomm Technologies, Inc, San Diego, CA, 92121, USA.; 3 Department of Industrial and Manufacturing Engineering, Pennsylvania State University, State College, PA, 16802 USA.

**Keywords:** Sparsity, Compressed sensing, Rank minimization, ADMM, System identification, Matrix completion, Proximal gradient method

## Abstract

Sparsity finds applications in diverse areas such as statistics, machine learning, and signal processing. Computations over sparse structures are less complex compared to their dense counterparts and need less storage. This paper proposes a heuristic method for retrieving sparse approximate solutions of optimization problems via minimizing the $\ell_{p}$ quasi-norm, where 0<p<1. An iterative two-block algorithm for minimizing the $\ell_{p}$ quasi-norm subject to convex constraints is proposed. The proposed algorithm requires solving for the roots of a scalar degree polynomial as opposed to applying a soft thresholding operator in the case of $\ell_{1}$ norm minimization. The algorithm’s merit relies on its ability to solve the $\ell_{p}$ quasi-norm minimization subject to any convex constraints set. For the specific case of constraints defined by differentiable functions with Lipschitz continuous gradient, a second, faster algorithm is proposed. Using a proximal gradient step, we mitigate the convex projection step and hence enhance the algorithm’s speed while proving its convergence. We present various applications where the proposed algorithm excels, namely, sparse signal reconstruction, system identification, and matrix completion. The results demonstrate the significant gains obtained by the proposed algorithm compared to other $\ell_{p}$ quasi-norm based methods presented in previous literature.

## 1 Introduction

### 1.1 Motivation

In numerical analysis and scientific computing, a sparse matrix/array is the one with many of its elements being zeros. The number of zeros divided by the total number of elements is called sparsity. Sparse data is often easier to store and process. Hence, techniques for deriving sparse solutions and exploiting them have attracted the attention of many researchers in various engineering fields like machine learning, signal processing, and control theory.

The taxonomy of sparsity can be studied through the Rank Minimization Problem (RMP). It has been lately considered in many engineering applications including control design and system identification. This is because the notions of complexity and system order can be closely related to the matrix rank. The RMP can be formulated as follows:

(1) $$\underset{X\in ℳ}{min}\;\;Rank(X)$$

where $X\in R^{m\times n}$ and $ℳ\subset R^{m\times n}$ is a convex set. The problem (1) in its generality is NP-hard [1]. Therefore, polynomial time algorithms for solving large-scale problems of the form in (1) are not currently known. Hence, recently adopted methods for solving such problems are approximate and structured heuristics. A special case of RMP is the Sparse Vector Recovery (SVR) problem involving $\ell_{0}$ pseudo-norm minimization given by:

(2) $$\underset{x\in 𝒱}{\text{min}}\;\;\;\;\;{\left\|x\right\|}_{0},$$

where $x\in R^{n}$, $𝒱\subset R^{n}$ is a closed convex set and $∥\cdot {∥}_{0}$ counts the number of the non-zero elements of its argument. From the definition of the rank being the number of non-zero singular values of a matrix, it can be easily realized that (1) is a generalized form of (2).

Numerous studies, which will be expounded upon in the subsequent section, have individually addressed effective solution methods for the problems presented in (1) and (2). These approaches utilize Schatten-p and $\ell_{p}$ quasi-norm relaxations, respectively. However, existing methods in this domain often either assume a predefined structure for the convex set ℳ in (1) or exclusively cater to the specialized case articulated in (2). Consequently, these methods lack comprehensive applicability. Leveraging the inherent relationship between the Schatten-p quasi-norm and the $\ell_{p}$ quasi-norm of matrix singular values, we endeavor to formulate an efficient heuristic method based on Schatten-p relaxation. This method is devised to address both problems in a unified manner. The proposed approach begins with the introduction of an algorithm for solving the $\ell_{p}$ quasi-norm relaxation of the SVR problem presented in (2). Subsequently, recognizing that (2) constitutes a specific case of (1), we utilize the developed $\ell_{p}$ quasi-norm minimization algorithm as a foundational component for constructing the envisaged generalized algorithm for RMPs.

### 1.2 Related Work

#### 1.2.1 Sparse vector recovery

Given that many signals exhibit sparsity or compressibility, the SVR problem has found widespread applications in fields such as object recognition, classification, and compressed sensing, as evidenced by studies such as [2, 3, 4]. The concept of sparse representation of signals and systems has been extensively discussed in [5], where the authors conducted a comprehensive review of both theoretical and empirical results pertaining to sparse optimization. They also derived the sufficient conditions necessary for ensuring uniqueness, stability, and computational feasibility. Moreover, [5] explores diverse applications of the SVR problem, contending that in certain tasks involving denoising and compression, methods rooted in sparse optimization offer state-of-the-art solutions.

The problem of constructing sparse solutions for undetermined linear systems has garnered significant attention. A survey conducted in [6] comprehensively examined existing algorithms for sparse approximation. The reviewed methods encompassed various approaches, including greedy methods [7, 8], techniques rooted in convex relaxation [3, 4], those employing non-convex optimization strategies [9, 10], and approaches necessitating brute force [11]. The authors discussed the computational demands of these algorithms and elucidated their interrelationships.

Sparse optimization problems of the form minf(x)+μg(x) have been extensively explored in the literature, where g(x) serves as a sparsity-inducing function, f represents a loss function capturing measurement errors, and μ>0 functions as a trade-off parameter balancing data fidelity and sparsity. In [12], the authors addressed a sparse recovery problem involving a set of corrupted measurements. By defining g(⋅) as the $\ell_{1}$ norm, they established a sufficient condition for exact sparse signal recovery, specifically the Restricted Isometry Property (RIP).

Motivated by the convergence of the $\ell_{p}$ quasi-norm to the $\ell_{0}$ pseudo-norm as p→0, the problem was extended in [13] by setting g as the $\ell_{p}$ quasi-norm for p∈(0,1). The authors presented theoretical results showcasing the $\ell_{p}$ quasi-norm’s capability to recover sparse signals from noisy measurements. Under more relaxed RIP conditions, it was demonstrated that the $\ell_{p}$ quasi-norm provides superior theoretical guarantees in terms of stability and robustness compared to $\ell_{1}$ minimization.

In [9], the authors considered the problem of SVR via $\ell_{p}$ quasi-norm minimization from a limited number of linear measurements of the target signal. However, the proposed approach faced limitations due to its higher computational complexity compared to the $\ell_{1}$ norm. In [14], Fourier-based algorithms for convex optimization were leveraged to solve sparse signal reconstruction problems via $\ell_{p}$ quasi-norm minimization, demonstrating a combination of the construction capabilities of nonconvex methods with the speed of convex ones.

An alternative approach for sparse reconstruction was proposed in [15], replacing the non-convex function with a quadratic convex one. Furthermore, [16] introduced an Alternating Direction Method of Multipliers (ADMM) [17] based algorithm enforcing both sparsity and group sparsity using non-convex regularization. Additionally, [18] proposed an iterative half-thresholding algorithm for expedited solutions of $\ell_{0.5}$ regularization. The authors not only established the existence of the resolvent of the gradient of the $\ell_{0.5}$ quasi-norm but also derived its analytic expression and provided a thresholding representation for the solutions. The convergence of this iterative half-thresholding algorithm was studied in [19], demonstrating its convergence to a local minimizer of the regularized problem with a linear convergence rate.

Conditions for the convergence of an ADMM algorithm aimed at minimizing the sum of a smooth function with a bounded Hessian and a non-smooth function are established in [20]. In [21], the convergence of ADMM is analyzed for the minimization of a non-convex and potentially non-smooth objective function subject to equality constraints. The derived convergence guarantee extends to various non-convex objectives, encompassing piece-wise linear functions, $\ell_{p}$ quasi-norm, and Schatten-p quasi-norm (0<p<1), while accommodating non-convex constraints. Several works have explored the $\ell_{1-2}$ relaxation objective, with [22] providing a theoretical analysis on SVR through weighted $\ell_{1-2}$ minimization when partial support information is available. Recovery conditions for exact SVR within a $\ell_{1-2}$ objective framework are derived in [23, 24], along with references therein, establishing the theoretical foundation for ensuring accurate SVR outcomes.

#### 1.2.2 Rank minimization

In [25], the authors sought to determine the least order dynamic output feedback, utilizing the formulation akin to (1), capable of stabilizing a linear time-invariant system. Their approach involved minimizing the trace, as opposed to the rank, resulting in a Semi-Definite Program (SDP) amenable to efficient solution techniques. Notably, their solution was specifically applicable to symmetric and square matrices. Building upon this work, [26] introduced a generalization of the aforementioned approach. This extension involved replacing the rank in the objective function with the summation of the singular values of the matrix, commonly known as the nuclear norm. The authors demonstrated that this modification yields the convex envelope of the non-convex rank objective, reducing to the original trace heuristic when the decision matrix assumes the form of a symmetric Positive Semi-Definite (PSD) matrix.

In [27], an alternative heuristic based on the logarithm of the determinant was introduced as a surrogate for rank minimization within the subspace of PSD matrices. The authors demonstrated that this formulation could be effectively solved through a sequence of trace minimization problems. In a related study, [28] delved into existing trace and log determinant heuristics, exploring their applications for computing a low-rank approximation in various scenarios. Specifically, the applications encompassed obtaining simple data models with interpretability by approximating *covariance matrices* for a given dataset.

Drawing inspiration from the success of the $\ell_{p}$ quasi-norm (0<p<1) for sparse signal reconstruction, an alternative method aims to enforce low-rank structure using the Schatten-p quasi-norm. This norm is defined as the $\ell_{p}$ quasi-norm of the singular values. In [29], the authors addressed the matrix completion problem, which involves constructing a low-rank matrix based on a subset of its entries. Instead of minimizing the nuclear norm, they proposed a Schatten- p quasi-norm formulation and investigated its convergence properties. To enhance the robustness of the solution, [30] combined the Schatten- p quasi-norm for low-rank recovery with the $\ell_{p}$ quasi-norm (0<p≤1) of prediction errors on the observed entries. The authors introduced an algorithm based on ADMM, which demonstrated superior numerical performance compared to other completion methods. In a non-convex approach for matrix optimization problems involving sparsity, [31] developed a technique using a generalized shrinkage operation. This method enhances the separation of moving objects from the stationary background by decomposing video into low-rank and sparse components, presenting advantages over the convex case.

### 1.3 Contributions

In spite of the commendable performance exhibited by the array of algorithms outlined in Sections 1.2.1 and 1.2.2, each designed to address different relaxations of (1) and (2), it is essential to acknowledge their problem-specific nature, primarily grounded in the specific structural attributes of the convex constraint sets they address. This issue of specialization results in a lack of generality across problem domains.

In this paper, we present a versatile algorithm grounded in the principles of projections onto constraint sets. A distinctive feature of this approach lies in its minimal reliance on problem-specific structural constraints, prioritizing the foundational characteristic of closed convexity. The works [32] and [33] delve into a comprehensive exploration, analyzing the intrinsic attributes of the projection operation onto constraint sets. While the former addresses the issue without incorporating a crucial coupling condition for polynomial equations, the latter assumes prior knowledge of the projection technique for each given point on $\ell_{p}$ balls.

Initially, we propose an ADMM algorithm, termed pQN-ADMM, designed to solve the $\ell_{p}$ quasi-norm relaxation of (2). At each iteration, the pivotal operation involves computing Euclidean projections onto specific convex and non-convex sets. Notably, the algorithm exhibits two key properties: 1) Its computational complexity aligns with that of $\ell_{1}$ minimization algorithms, with the additional task of solving for the roots of a polynomial; 2) It does not necessitate a specific structure for the convex set.

Subsequently, we extend the application of the proposed algorithm to address the relaxation of (1) by embracing the Schatten- p quasi-norm. In this extension, we leverage the equivalence between minimizing the $\ell_{p}$ quasi-norm of the vector of singular values and minimizing the Schatten- p quasi-norm. Our study encompasses the following numerical instances:

1. An example employing SVR, wherein the primary objective is the recovery of the sparsest feasible vector from given realizations.
2. A matrix completion example, where the overarching goal is the reconstruction of an unknown low-rank matrix based on a limited subset of observed entries.
3. Addressing a time-domain system identification problem, specifically tailored for minimum-order system detection.

Our numerical results compellingly showcase the competitiveness of pQN-ADMM when bench-marked against several state-of-the-art baseline methods.

Conclusively, given the inherent reliance of the derived algorithm on a convex projection step in each iteration, our endeavor is directed towards the formulation of an expedited algorithm accompanied by a rigorous mathematical convergence guarantee. Focusing on a subset of problems where the constraint set manifests as a polytope, we leverage principles from the Proximal Gradient (PG) method to formulate a rapid algorithm. The convergence of this algorithm is established with a rate of $O\left(\frac{1}{K}\right)$, where K denotes the iteration budget assigned to the algorithm.

## 2 Notation

Unless otherwise specified, we denote vectors with lowercase boldface letters, i.e., x, with i-th entry as $x_{i}$, while matrices are in uppercase, i.e. X, with (i,j)-th entry as $x_{i,j}$. For an integer $n\in Z_{+}$, [n]≜{1,…,n}. **1** represents a vector of all entries equal to 1, while $1_{𝒢}(.)$ is an indicator function to the set 𝒢, i.e., it evaluates to zero if its argument belongs to the set 𝒢 and is +∞ otherwise.

For a vector $x\in R^{n}$, the general $\ell_{p}$ norm is defined as:

(3) $${\left\|x\right\|}_{p}≜{\left(\sum_{i\in \left[n\right]}{\left|x_{i}\right|}^{p}\right)}^{\frac{1}{p}},$$

where, we let ∥x∥ be the well-known Euclidean norm, i.e., p=2. When 0<p<1, the expression in (3) is termed as a quasi-norm satisfying the same axioms of the norm except the triangular inequality making it a non-convex function.

For a matrix X, ∥X∥ represents the spectral norm, which is defined as the square root of the maximum eigenvalue of the matrix $X^{H}X$. $X^{H}$ refers to the complex conjugate transpose of X, denoted as $X^{⊤}$. On the other hand, $∥\cdot {∥}_{f}$ signifies the Frobenius norm of a matrix.

The Schatten-p quasi-norm of a matrix X is defined as:

(4) $${\left\|X\right\|}_{p,*}≜{\left(\sum_{i\in \left[\text{min}\left\{m,n\right\}\right]}\sigma_{i}{\left(X\right)}^{p}\right)}^{\frac{1}{p}},$$

where $\sigma_{i}(X)$ is the i-th singular value of the matrix X. We utilize the * subscript in (4) to differentiate the matrix Schatten- p quasi-norm from vector $\ell_{p}$ case defined in (3).

When p=1, (4) yields the nuclear norm which is the convex envelope of the rank function. Throughout the paper, we consider a non-convex relaxation for the rank function, specifically p=1/2.

We define ⌈⋅⌉ as the ceiling operator, $vec(X)\in R^{mn}$ as a vector formed by stacking the columns of the matrix $X\in R^{m\times n}$ and Hankel(.) as an operator that outputs a Hankel matrix constructed from the applied vector arguments.

## 3 Sparse Vector Recovery Algorithm

### 3.1 Problem Formulation

This section develops a method for approximating the solution of (2) using the following relaxation:

(5) $$\underset{x\in 𝒱}{\text{min}}\;\;\;{\left\|x\right\|}_{p}^{p},$$

where p∈(0,1] and 𝒱 is a closed convex set. Problem (5) is convex for p≥1; hence, can be solved to optimality efficiently. However, the problem becomes non-convex when p<1. We present a gradient-based algorithm and consequently, it may not always converge to a global optimum solution but only to a stationary point. An epigraph equivalent formulation of (5) is obtained by introducing the variable $t={\left[t_{i}\right]}_{i\in [n]}:$

(6) $$\underset{x,t}{min}\;1^{⊤}t,\;\text{s.t.}t_{i}\geq {\left|x_{i}\right|}^{p},\;i\in [n],\;x\in 𝒱.$$

Let $𝒳\subset R^{2}$ denote the epigraph of the scalar function $|x{|}^{p}$, i.e., $𝒳=\{(x,t)\in R^{2}\;:t\geq |x{|}^{p}\}$, which is a non-convex set for p<1. Then, (6) can be cast as:

(7) $$\underset{x,t}{\text{min}}\sum_{i\in \left[n\right]}1_{𝒳}\left(x_{i},t_{i}\right)+1^{⊤}t,\;\text{s}.t.\;x\in 𝒱.$$

ADMM, as introduced in [17], leverages the inherent problem structure to partition the optimization process into simpler sub-problems, which are solved iteratively. To achieve this, auxiliary variables $y={\left[y_{i}\right]}_{i\in [n]}$ and $z={\left[z_{i}\right]}_{i\in [n]}$ are introduced, leading to an ADMM reformulation of the problem defined in (7):

(8) $$\underset{x,t,y,z}{\text{min}}\;\;\;\;\sum_{i\in \left[n\right]}1_{𝒳}\left(x_{i},t_{i}\right)+1_{𝒱}\left(y\right)+1^{⊤}z,\;s\text{.t}.\;x=y:\lambda ,\;t=z:\theta .$$

The dual variables associated with the constraints x=y and t=z are λ and θ, respectively. Throughout the paper, the colons in the constraints of an optimization problem serve as a means to associate the constraint (appearing on the left side of the colon) with its corresponding Lagrange multiplier (found on the right side of the colon). The Lagrangian function corresponding to (8) augmented with a quadratic penalty on the violation of the equality constraints with penalty parameter ρ>0, is given by:

(9) $${ℒ}_{\rho }\left(x,t,y,z,\lambda ,\theta \right)=\sum_{i\in \left[n\right]}1_{𝒳}\left(x_{i},t_{i}\right)+1_{𝒱}\left(y\right)+1^{⊤}z\;+\lambda^{⊤}(x-y)+\theta^{⊤}(t-z)+\frac{\rho }{2}\left(\|x-y{\|}^{2}+\|t-z{\|}^{2}\right).$$

Considering the two block variables (x,t) and (y,z), ADMM consists of the following iterations:

(10) $$(x,t{)}^{k+1}=\underset{x,t}{argmin}{\;ℒ}_{\rho }\left(x,t,y^{k},z^{k},\lambda^{k},\theta^{k}\right),$$

(11) $$(y,z{)}^{k+1}=\underset{y,z}{argmin}{\;ℒ}_{\rho }\left(x^{k+1},t^{k+1},y,z,\lambda^{k},\theta^{k}\right),$$

(12) $$\lambda^{k+1}=\lambda^{k}+\rho \left(x^{k+1}-y^{k+1}\right),$$

(13) $$\theta^{k+1}=\theta^{k}+\rho \left(t^{k+1}-z^{k+1}\right).$$

Given the augmented Lagrangian function expressed in (9), it is evident from (10) that the variables x and t are iteratively updated by solving the following non-convex problem:

**Algorithm 1:** ADMM (ρ>0)

1:	Initialize: $y^{0}$, $z^{0}$, $\lambda^{0}$, $\theta^{0}$
2:	**for** k≥0 **do**
3:	${\left(x_{i},t_{i}\right)}^{k+1}\leftarrow \Pi_{𝒳}\left(y_{i}^{k}-\frac{\lambda_{i}^{k}}{\rho },z_{i}^{k}-\frac{\theta_{i}^{k}}{\rho }\right),\forall i\in [n]$
4:	$y^{k+1}\leftarrow \Pi_{𝒴}\left(x^{k+1}+\frac{\lambda^{k}}{\rho }\right)$
5:	$z^{k+1}\leftarrow t^{k+1}+\frac{\theta^{k}-1}{\rho }$
6:	$\lambda^{k+1}\leftarrow \lambda^{k}+\rho \left(x^{k+1}-y^{k+1}\right)$
7:	$\theta^{k+1}\leftarrow \theta^{k}+\rho \left(t^{k+1}-z^{k+1}\right)$


(14) $$\underset{x,t}{min}\;{∥x-y^{k}+\frac{\lambda^{k}}{\rho }∥}^{2}+{∥t-z^{k}+\frac{\theta^{k}}{\rho }∥}^{2},\;\text{s.t.}\left(x_{i},t_{i}\right)\in 𝒳,\;i\in [n].$$

Exploiting the separable structure of (14), one immediately concludes that (14) can be split into n independent 2-dimensional problems that can be solved in parallel, i.e., for each i∈[n]:

(15) $${\left(x_{i},t_{i}\right)}^{k+1}=\Pi_{𝒳}\left(y_{i}^{k}-\frac{\lambda_{i}^{k}}{\rho },z_{i}^{k}-\frac{\theta_{i}^{k}}{\rho }\right),$$

where $\Pi_{𝒳}(.)$ denotes the Euclidean projection operator onto the set X. Furthermore, (9) and (11) imply that y and z are independently updated as follows:

(16) $$y^{k+1}=\Pi_{𝒱}\left(x^{k+1}+\frac{\lambda^{k}}{\rho }\right),$$

(17) $$z^{k+1}\;=t^{k+1}+\frac{\theta^{k}-1}{\rho }.$$

Algorithm 1 summarizes the proposed ADMM algorithm. It is clear that z, λ, and θ merit closed-form updates. However, updating (x,t) requires solving n non-convex problems. Our strategy for dealing with this issue is presented in the following section.

### 3.2 Non-convex Projection

In this section, we present the method used to tackle the non-convex projection problem required to update x and t.

Among the advantages of the proposed algorithm is that it is amenable to de-centralization. As it is clear from (15), x and t can be updated element-wise via performing a projection operation onto the non-convex set 𝒳, one for each i∈[n]. The n projection problems can be run independently in parallel. We now outline the proposed idea for solving one such projection, i.e., we suppress the dependence on the index of the entry of x and t. For $(\overset{‾}{x},\overset{‾}{t})\in R^{2}$, $\Pi_{𝒳}(\overset{‾}{x},\overset{‾}{t})$ entails solving:

(18) $$\underset{x,t}{min}\;\;g(x,t)≜(t-\overset{‾}{t}{)}^{2}+(x-\overset{‾}{x}{)}^{2},\;\text{s.t.}\;t\geq |x{|}^{p}.$$

If $\overset{‾}{t}\geq |\overset{‾}{x}{|}^{p}$, then trivially $\Pi_{𝒳}(\overset{‾}{x},\overset{‾}{t})=(\overset{‾}{x},\overset{‾}{t})$. Thus, we focus on the case in which $\overset{‾}{t}<|\overset{‾}{x}{|}^{p}$. The following theorem states the necessary optimality conditions for (18).

#### Theorem 1

*Let* $\overset{‾}{t}<|\overset{‾}{x}{|}^{p}$, *and* $\left(x^{*},t^{*}\right)$ *be an optimal solution of* (18). *Then, the following properties are satisfied:*

- $sign\left(x^{*}\right)=sign(\overset{‾}{x})$,
-
$t^{*}\geq \overset{‾}{t}$

-
${\left|x^{*}\right|}^{p}\geq \overset{‾}{t}$

- $t^{*}={\left|x^{*}\right|}^{p}$.

##### Proof

We prove the statements by contradiction as follows:

- Suppose that $sign\left(x^{*}\right)\neq sign(\overset{‾}{x})$, then:

(19) $$\left|x^{*}-\overset{‾}{x}\right|=\left|x^{*}-0\right|+|\overset{‾}{x}-0|>|\overset{‾}{x}-0|,$$

i.e., ${\left(x^{*}-\overset{‾}{x}\right)}^{2}>(0-\overset{‾}{x}{)}^{2}$. Hence, $g\left(x^{*},t^{*}\right)-g\left(0,t^{*}\right)>0$. Moreover, the feasibility of $\left(x^{*},t^{*}\right)$ implies that $t^{*}>0$. Thus, $\left(0,t^{*}\right)$ is feasible and attains a lower objective value than that attained by $\left(x^{*},t^{*}\right)$. This contradicts the optimality of $\left(x^{*},t^{*}\right)$.

- Assume that $t^{*}<\overset{‾}{t}$. Then:

(20) $$g\left(x^{*},t^{*}\right)-g\left(x^{*},\overset{‾}{t}\right)={\left(t^{*}-\overset{‾}{t}\right)}^{2}>0.$$

Furthermore, by the feasibility of $\left(x^{*},t^{*}\right)$, we have ${\left|x^{*}\right|}^{p}\leq t^{*}<\overset{‾}{t}$. Thus, $\left(x^{*},\overset{‾}{t}\right)$ is feasible and attains a lower objective value than that attained by $\left(x^{*},t^{*}\right)$. This contradicts the optimality of $\left(x^{*},t^{*}\right)$.

- Suppose that ${\left|x^{*}\right|}^{p}<\overset{‾}{t}$, i.e.,

(21) $$-{\overset{‾}{t}}^{\frac{1}{p}}<x^{*}<{\overset{‾}{t}}^{\frac{1}{p}}.$$

We now consider two cases, $\overset{‾}{x}>0$ and $\overset{‾}{x}<0$. First, let $\overset{‾}{x}>0$. Then, we have by (a) and (21) that $0<x^{*}<{\overset{‾}{t}}^{\frac{1}{p}}$. Since $\overset{‾}{t}<|\overset{‾}{x}{|}^{p}$, i.e., $(\overset{‾}{x},\overset{‾}{t})\notin 𝒳$, therefore ${\overset{‾}{t}}^{\frac{1}{p}}<\overset{‾}{x}$ and hence, $0<x^{*}<{\overset{‾}{t}}^{\frac{1}{p}}<\overset{‾}{x}$. Pick $x_{0}>0$ such that ${\left|x_{0}\right|}^{p}=\overset{‾}{t}$, i.e., $x_{0}={\overset{‾}{t}}^{\frac{1}{p}}$. Then clearly, $x^{*}<x_{0}<\overset{‾}{x}$. Thus, we have:

(22) $$g\left(x^{*},t^{*}\right)-g\left(x_{0},t^{*}\right)={\left(x^{*}-\overset{‾}{x}\right)}^{2}-{\left(x_{0}-\overset{‾}{x}\right)}^{2}>0,$$

where the last inequality follows the just proven identity that $x^{*}<x_{0}<\overset{‾}{x}$. Moreover, we have ${\left|x_{0}\right|}^{p}=\overset{‾}{t}\leq t^{*}$ by (*b*). Thus, $\left(x_{0},t^{*}\right)$ is feasible and attains a lower objective value than that attained by $\left(x^{*},t^{*}\right)$. This contradicts the optimality of $\left(x^{*},t^{*}\right)$. On the other hand, let $\overset{‾}{x}<0$. Then, we have by (*a*) and (21) that $-{\overset{‾}{t}}^{\frac{1}{p}}<x^{*}<0$. Since $\overset{‾}{t}<|\overset{‾}{x}{|}^{p}$, i.e., $(\overset{‾}{x},\overset{‾}{t})\notin 𝒳$, then ${\overset{‾}{t}}^{\frac{1}{p}}<|\overset{‾}{x}|$, i.e., $\overset{‾}{x}<-{\overset{‾}{t}}^{\frac{1}{p}}$. Therefore, $\overset{‾}{x}<-{\overset{‾}{t}}^{\frac{1}{p}}<x^{*}$. Pick $x_{0}<0$ such that ${\left|x_{0}\right|}^{p}=\overset{‾}{t}$, i.e., $x_{0}=-{\overset{‾}{t}}^{\frac{1}{p}}$. Then, (22) also holds when $\overset{‾}{x}<0$. Note that ${\left|x_{0}\right|}^{p}=\overset{‾}{t}\leq t^{*}$ by (*b*). Thus, $\left(x_{0},t^{*}\right)$ is feasible and attains a lower objective value than that attained by $\left(x^{*},t^{*}\right)$. This contradicts the optimality of $\left(x^{*},t^{*}\right)$.

- The feasibility of $\left(x^{*},t^{*}\right)$ eliminates the possibility that $t^{*}<{\left|x^{*}\right|}^{p}$. Now let $t^{*}>{\left|x^{*}\right|}^{p}$ and pick $t_{0}={\left|x^{*}\right|}^{p}$. Then, $\overset{‾}{t}\leq {\left|x^{*}\right|}^{p}=t_{0}<t^{*}$, where the first inequality follows from (*c*). Then, $0\leq t_{0}-\overset{‾}{t}<t^{*}-\overset{‾}{t}$. Thus, we have:

(23) $$g\left(x^{*},t^{*}\right)-g\left(x^{*},t_{0}\right)={\left(t^{*}-\overset{‾}{t}\right)}^{2}-{\left(t_{0}-\overset{‾}{t}\right)}^{2}>0,$$

Furthermore, the feasibility of $\left(x^{*},t_{0}\right)$ follows trivially from the choice of $t_{0}$. Thus, $\left(x^{*},t_{0}\right)$ is feasible and attains a lower objective value than that attained by $\left(x^{*},t^{*}\right)$. This contradicts the optimality of $\left(x^{*},t^{*}\right)$.

This concludes the proof.

We now make use of the fact that for (18), an optimal solution $\left(x^{*},t^{*}\right)$ satisfies $t^{*}={\left|x^{*}\right|}^{p}$ and hence, (18) reduces to solving:

(24) $$\underset{x}{min}\;{\left(|x{|}^{p}-\overset{‾}{t}\right)}^{2}+(x-\overset{‾}{x}{)}^{2}.$$

**Algorithm 2:** Non-convex projection $\left(p=\frac{s}{q}<1\right)$

1:	$ℛ\leftarrow roots\{a^{2q}+\frac{s}{q}\left(a^{2s}-\overset{‾}{t}a^{s}\right)-|\overset{‾}{x}|a^{q}\}$
2:	$\bar{ℛ}\leftarrow ℛ\backslash \{complex\;numbers\;and\;negative\;reals\;in\;ℛ\}$
3:	$𝒯\leftarrow \{\left(r^{q},r^{s}\right)\;:r\in \bar{ℛ}\}$
4:	$\left(\overset{ˆ}{x},t^{*}\right)\leftarrow argmin\{g(x,t)\;:(x,t)\in 𝒯\}$
5:	$x^{*}\leftarrow sign(\overset{‾}{x})\overset{ˆ}{x}$

The first order necessary optimality condition for (24) implies the following:

(25) $$p{\left|x^{*}\right|}^{p-1}sign\left(x^{*}\right)\left({\left|x^{*}\right|}^{p}-\overset{‾}{t}\right)+x^{*}-\overset{‾}{x}=0.$$

By the symmetry of the function $|x{|}^{p}$, without loss of generality, assume that $x^{*}>0$ and let $0<p=\frac{s}{q}<1$ for some $s,q\in Z_{+}$. A change of variables $a^{q}=x^{*}$ plugged in (25) shows that finding an optimal solution for (18) reduces to finding a root of the following scalar degree 2q polynomial:

(26) $$a^{2q}+\frac{s}{q}\left(a^{2s}-\overset{‾}{t}a^{s}\right)-\overset{‾}{x}a^{q}.$$

To determine $\Pi_{𝒳}(\overset{‾}{x},\overset{‾}{t})$, the objective is to find a root denoted as $a^{*}$ for the polynomial in (26), while ensuring that the pair $\left(a^{{*}^{q}},a^{{*}^{s}}\right)$ minimizes the function g(x,t). Algorithm 2 provides a summary of the method employed to address problem (18). When both $x^{*}=0$ and $t^{*}=0$, the objective function evaluates to $g(0,\;0)={\overset{‾}{x}}^{2}+{\overset{‾}{t}}^{2}$. To optimize the objective function while upholding the constraint $t\geq |x{|}^{p}$, the choice is made to set $x^{*}=0$ and $t^{*}=max\{0,\;\overset{‾}{t}\}$. This decision ensures that $g\left(0,t^{*}\right)\leq g(0,\;0)$. In instances where $\overset{‾}{x}=0$ and $\overset{‾}{t}\leq |\overset{‾}{x}{|}^{p}$, indicating that $\overset{‾}{t}\leq 0$, the choice is to set $x^{*}=0$. Consequently, this results in $g(0,t)=(t-\overset{‾}{t}{)}^{2}=(t+|\overset{‾}{t}|{)}^{2}$. To meet the constraint $t^{*}\geq {\left|x^{*}\right|}^{p}$, the optimal selection is $t^{*}=0$, which stands as the most suitable option for minimizing g(0,t).

### 3.3 Convex Projection

The convex projection for y-update in (16) can be formulated as the following convex optimization problem:

(27) $$y^{k+1}=\underset{y\in 𝒱}{argmin}{\;∥y-(x^{k+1}+\frac{\lambda^{k}}{\rho })∥}^{2}.$$

Convex problems can be solved by a variety of contemporary methods including bundle methods [34], sub-gradient projection [35], interior point methods [36], and ellipsoid methods [37]. The efficiency of optimization techniques relies mainly on exploiting the structure of the constraint set. As discussed in Section 1.3, our objective is to address the problem outlined in (5) with minimal assumptions on the set 𝒱. Our only requirement is that 𝒱 is a closed and convex set. Nevertheless, if feasible, one should capitalize on the inherent structure of 𝒱 to potentially streamline the computational complexity involved in solving (27).

## 4 Rank Minimization Algorithm

We consider the problem in (1) and propose a method for approximating its solution efficiently. The Schatten- p heuristic of (1) can be written as:

(28) $$\underset{X\in ℳ}{\text{min}}\;\;\;\;{\left\|X\right\|}_{p,*}^{p}≜\sum_{i=1}^{\text{L}}{\left|\sigma_{i}\left(X\right)\right|}^{p},$$

where L=min{m,n} and $\sigma_{i}(X)$ is the i th singular value of X. In the scenario where p=1, (28) represents a convex problem, akin to the nuclear norm heuristic. We now consider a non-convex relaxation, specifically for the case where 0<p<1. The problem in (28) attains an epi-graph form:

(29) $$\underset{X,t}{min}\;1^{⊤}t,\;\text{s.t.}{\left|\sigma_{i}(X)\right|}^{p}\leq t_{i},\;i\in \left\{1,\ldots L\right\},\;X\in ℳ,$$

such that $t={\left[t_{i}\right]}_{i\in [L]}$. Defining the epi-graph set 𝒴 for the function σ(X), where $𝒴≜\{(\sigma (X),t)\in R^{2}:|\sigma (X){|}^{p}\leq t\}\subseteq R^{2}$, the problem in (29) can be written as:

(30) $$\underset{X,t}{\text{min}}\;\;\;1^{⊤}t+1_{ℳ}\left(X\right)+\sum_{i=1}^{\text{L}}1_{𝒴}\left(\sigma_{i}\left(X\right),t_{i}\right).$$

To formulate the problem in a manner amenable to ADMM, we introduce auxiliary variables, $Y\in R^{m\times n}$ and $z={\left[z_{i}\right]}_{i\in [L]}$. This transformation leads to the following representation of the problem in (30):

(31) $$\underset{X,t,Y,z}{\text{min}}\;\;\;\;1^{⊤}z+1_{ℳ}\left(Y\right)+\sum_{i=1}^{\text{L}}1_{𝒴}\left(\sigma_{i}\left(X\right),t_{i}\right),\;\;\;\;\;s\text{.t}.\;\;\;\;\;\;\;\;\;\;\;\;\;X=Y:\Lambda ,\;\;\;t=z:\theta ,$$

where Λ, θ are the dual variables associated with X and t respectively. Similar to (9), the Lagrangian function associated with (31), augmented with a quadratic penalty for the equality constraint violation with a parameter ρ>0, can be represented as:

(32) $${ℒ}_{\rho }\left(X,Y,t,z,\Lambda ,\theta \right)=1^{⊤}z+1_{ℳ}\left(Y\right)+\sum_{i=1}^{\text{L}}1_{𝒴}\left(\sigma_{i}\left(X\right),t_{i}\right)\;+Tr\left\{\Lambda^{⊤}(X-Y)\right\}+\theta^{⊤}(t-z)+\frac{\rho }{2}\left(\|X-Y{\|}_{\text{f}}^{2}+\|t-z{\|}^{2}\right),$$

where Tr{.} is the trace operator. Given the 2-tuples (X,t) and (Y,z), the ADMM iterations are as follows:

(33) $$(X,t{)}^{k+1}=\underset{X,t}{argmin}\;{ℒ}_{\rho }\left(X,Y^{k},t,z^{k},\Lambda^{k},\theta^{k}\right),$$

(34) $$Y^{k+1}=\underset{Y}{argmin}\;{ℒ}_{\rho }\left(X^{k+1},Y,t^{k+1},z^{k},\Lambda^{k},\theta^{k}\right),$$

(35) $$z^{k+1}=\underset{z}{argmin}\;{ℒ}_{\rho }\left(X^{k+1},Y^{k+1},t^{k+1},z,\Lambda^{k},\theta^{k}\right),$$

(36) $$\Lambda^{k+1}=\Lambda^{k}+\rho \left(X^{k+1}-Y^{k+1}\right),$$

(37) $$\theta^{k+1}=\theta^{k}+\rho \left(t^{k+1}-z^{k+1}\right).$$

### 4.1 (X,t) Update

By completing the square and employing some straightforward algebraic manipulations, it can be demonstrated that the problem described in (33) is equivalent to:

(38) $$\underset{X,t}{min}\;{∥X-{\bar{X}}^{k}∥}_{f}^{2}+{∥t-{\bar{t}}^{k}∥}^{2},\;\text{s.t.}{\left|\sigma_{i}(X)\right|}^{p}\leq t_{i},\;\;i\in \left\{1,\ldots L\right\},$$

where ${\bar{X}}^{k}≜Y^{k}-\frac{\Lambda^{k}}{\rho }$ and ${\bar{t}}^{k}≜z^{k}-\frac{\theta^{k}}{\rho }$. For simplicity, we will omit the iteration index k. Let’s assume that $X=P\Sigma Q^{⊤}$ and $\bar{X}=U\Delta V^{⊤}$ represent the Singular Value Decomposition (SVD) of X and $\bar{X}$, respectively. Here, Σ and Δ are diagonal matrices with the singular values associated with X and $\bar{X}$, while P, U, Q, and V are unitary matrices. Following the steps in [38, Theorem 3], we can express the first term of (38) as:

(39) $$∥X-\bar{X}{∥}_{f}^{2}={∥P\Sigma Q^{⊤}-U\Delta V^{⊤}∥}_{f}^{2}\;\;={∥P\Sigma Q^{⊤}∥}_{f}^{2}+{∥U\Delta V^{⊤}∥}_{f}^{2}-2T\left\{X^{⊤}\bar{X}\right\}\;\;\overset{\left(a\right)}{=}Tr\left\{\Sigma^{⊤}\Sigma \right\}+Tr\left\{\Delta^{⊤}\Delta \right\}-2Tr\left\{Q\Sigma^{⊤}P^{⊤}U\Delta V^{⊤}\right\}\;\;\overset{\left(b\right)}{\geq }Tr\left\{\Sigma^{⊤}\Sigma \right\}+Tr\left\{\Delta^{⊤}\Delta \right\}-2Tr\left\{\Sigma^{⊤}\Delta \right\}=∥\Sigma -\Delta {∥}_{f}^{2},$$

where (a) is because $P^{⊤}P=Q^{⊤}Q=U^{⊤}U=V^{⊤}V=I_{L\times L}$ with $I_{L\times L}$ being an identity matrix of size L, and exploiting the circular property of the trace while (b) holds is from the main result of [39]. In order to make ${∥X-{\bar{X}}^{k}∥}_{f}^{2}$ achieve its derived lower bound, we set P=U and Q=V.

The problem in (38) is then equivalent to:

(40) $$\begin{array}{ll} \underset{x,t}{min}\; & ∥x-\bar{x}{∥}^{2}+∥t-\bar{t}{∥}^{2},\\ \text{s.t.} & {\left|x_{i}\right|}^{p}\leq t_{i},\;i\in \left\{1,\ldots L\right\}, \end{array}$$

where $x={\left[x_{i}\right]}_{i\in [L]}$ and $\bar{x}={\left[{\overset{‾}{x}}_{i}\right]}_{i\in [L]}$ are the vectors of singular values of the matrices X and $\bar{X}$ respectively. The optimal solution $X^{*}$ for (38) can be determined by first finding the optimal $x^{*}$ for (40), and then obtaining $X^{*}=U\Sigma^{*}V^{⊤}$, where $\Sigma^{*}=diag\left(x^{*}\right)$ and diag(.) denotes an operator that transforms a vector into its corresponding diagonal matrix. Given that the problem in (40) is separable, we will proceed by omitting the index i and focus solely on solving:

(41) $$\underset{x,t}{min}\;\;\;(x-\overset{‾}{x}{)}^{2}+(t-\overset{‾}{t}{)}^{2},\;\text{s.t.}\;|x{|}^{p}\leq t.$$

It can be realized that (41) is similar to (18), hence, its optimal solution can be found by applying Algorithm 2.

### 4.2 (Y,z) Update

Upon updating (X,t) with Λ and θ held constant, the problem in (34) can be reformulated as:

(42) $$Y^{k+1}=\underset{Y\in ℳ}{argmin}{∥Y-\left(X^{k+1}+\frac{\Lambda^{k}}{\rho }\right)∥}_{f}^{2},$$

which is clearly a convex optimization problem representing the projection of the point $X^{k+1}+\frac{\Lambda^{k}}{\rho }$ on the set ℳ and can be solved by various known class of algorithms as discussed in Section 3.3. Following the update of Y, the update for z in (35) is as follows:

(43) $$z^{k+1}=\underset{z}{argmin\;}1^{⊤}z+\frac{\rho }{2}{∥z-\left(t^{k+1}+\frac{\theta^{k}}{\rho }\right)∥}^{2},$$

which results in a closed-form solution for $z^{k+1}=t^{k+1}+\frac{\theta^{k}-1}{\rho }$.

## 5 Proximal Gradient Algorithm

The pQN-ADMM algorithm adeptly handles the $\ell_{p}$ relaxation of (2), refraining from assuming any specific structure for 𝒱 beyond its closed and convex nature. Primarily, the algorithm hinges on the computation of Euclidean projections onto 𝒱, as outlined in (27).

In this section, we consider a sub-class of problems with a specific structure for the convex set of the form 𝒱={x:f(x)≤0}, where f(x) is a convex function with Lipschitz continuous gradient. i.e., f is L-smooth: ∥∇f(x)−∇f(y)∥≤L∥x−y∥ for all $x,\;y\in R^{n}$. Specifically, in order to solve:

(44) $$\underset{x}{min}\;∥x{∥}_{p}^{p},\;\text{s.t.}\;f(x)\leq 0,$$

we aim to develop an efficient algorithm with some convergence guarantees for the following Lagrangian relaxation:

(45) $$\underset{x}{min}\;\;F(x)≜∥x{∥}_{p}^{p}+\frac{\mu }{2}f(x),$$

where μ≥0 is the dual multiplier that captures the trade-off between solution sparsity and fidelity. It is imperative to acknowledge that (44) and (45) exhibit a relationship, albeit not being strictly equivalent.

A canonical problem for the regularized risk minimization has the following form:

(46) $$\underset{x}{min}\;g\left(x\right)+h\left(x\right),$$

where h is an L-smooth loss function, and g represents the regularizer term. In cases where both g and h exhibit convexity, the Proximal Gradient (PG) algorithm [40] can iteratively compute a solution to (46) through PG steps.

(47) $$x^{k+1}={prox}_{g/\lambda }\left(x^{k}-\nabla h\left(x^{k}\right)/L\right),$$

where ${prox}_{g/\lambda }(.)≜{argmin}_{x}g(x)+\frac{\lambda }{2}∥x-\cdot {∥}^{2}$, for some constant λ. When g is convex, the proximal map ${prox}_{g/\lambda }$ is well-defined, thus, the PG step can be computed. In comparing both (45) and (46), it is observed that the convexity assumption of g(x) in (46) is not met for $∥x{∥}_{p}^{p}$ in (45). When the regularizer is a continuous non-convex function, the proximal map ${prox}_{g/\lambda }$ may not exist, and computing it in closed form becomes a challenging task.

On the contrary, in the case of $∥x{∥}_{p}^{p}$, leveraging similar reasoning as employed in the non-convex projection step introduced in Section 3.2, our objective is to derive an analytical solution that can be efficiently computed. Specifically, assuming p∈(0,1) is a positive rational number, the proposed method for computing the proximal map of $∥x{∥}_{p}^{p}$ involves finding the roots of a polynomial of order 2q, where $q\in Z_{+}$ such that p=s/q for some $s\in Z_{+}$.

Since f is L-smooth, for all $x,y\in R^{n}$, we have:

(48) $$f(x)\leq f(y)+\nabla f(y{)}^{⊤}(x-y)+\frac{L}{2}∥x-y{∥}^{2}.$$

Given $x^{k}$, replacing f(x) with the upper bound in (48) for $y=x^{k}$, the prox-gradient operation naturally arises as follows:

(49) $$x^{k+1}=\underset{x}{argmin}∥x{∥}_{p}^{p}+\frac{\mu }{2}\left[f\left(x^{k}\right)+\nabla f{\left(x^{k}\right)}^{⊤}\left(x-x^{k}\right)+\frac{L}{2}{∥x-x^{k}∥}^{2}\right].$$

By completing the square, (49) yields to:

(50) $$x^{k+1}=\underset{x}{argmin}∥x{∥}_{p}^{p}+\frac{\mu L}{4}{∥x-\left(x^{k}-\frac{1}{L}\nabla f\left(x^{k}\right)\right)∥}^{2}.$$

Defining ${\bar{x}}^{k}≜x^{k}-\frac{1}{L}\nabla f\left(x^{k}\right)$, (50) can be rewritten as:

(51) $$x^{k+1}=\underset{x}{\mathrm{argmin}}\|x{\|}_{p}^{p}+\frac{\mu L}{4}{\left\|x-{\bar{x}}^{k}\right\|}^{2}\;\;\;\;\;\;\;\;\;\;\;=\underset{x}{\mathrm{argmin}}\sum_{i=1}^{n}{\left|x_{i}\right|}^{p}+\frac{\mu L}{4}{\left(x_{i}-{\bar{x}}_{i}^{k}\right)}^{2},$$

which is clearly a separable structure in the entries of x. Therefore, for each i∈[n], we have:

(52) $$x_{i}^{k+1}=\underset{x_{i}}{argmin}{\left|x_{i}\right|}^{p}+\frac{\mu L}{4}{\left(x_{i}-{\overset{‾}{x}}_{i}^{k}\right)}^{2}={prox}_{\overset{‾}{g}/\frac{\mu L}{2}}\left({\overset{‾}{x}}_{i}^{k}\right),$$

where $\overset{‾}{g}\;:R\to R_{+}$ such that $\overset{‾}{g}(t)=|t{|}^{p}$ for some positive rational p∈(0,1).

Next, we consider a generic form of (52), i.e., given some $\overset{‾}{t}\in R$, we would like to compute:

(53) $$t^{*}=\underset{t}{argmin}\{|t{|}^{p}+\frac{\mu L}{4}(t-\overset{‾}{t}{)}^{2}\}.$$

The first-order optimality condition for (53) can be written as:

(54) $$p{\left|t^{*}\right|}^{p-1}sign\left(t^{*}\right)+\frac{\mu L}{2}\left(t^{*}-\overset{‾}{t}\right)=0.$$

Using similar arguments as in Section 3.2, we can conclude that the optimal solution $t^{*}$ attains the property that $sign\left(t^{*}\right)=sign(\overset{‾}{t})$. Without loss of generality, exploiting the symmetry of the function $\overset{‾}{g}$, we only consider the case when $\overset{‾}{t}>0$; hence, the optimal solution $t^{*}$ is the smallest positive root of the following polynomial:

(55) $$p{\left|t^{*}\right|}^{p-1}+\frac{\mu L}{2}\left(t^{*}-\overset{‾}{t}\right)=0.$$

Similar to (26), suppose $0<p=\frac{s}{q}<1$ for some positive integers s and q. By employing the variable transformation $a≜{\left(t^{*}\right)}^{\frac{1}{q}}$, the optimality condition in (55) is simplified to the task of finding the roots of a polynomial of degree 2q:

(56) $$a^{2q}-\overset{‾}{t}a^{q}+\frac{2s}{q\mu L}a^{s}=0.$$

In order to solve (44) effectively, we will employ Algorithm 3, which implements the non-convex inexact Accelerated Proximal Gradient (APG) descent method as presented in [41, Algorithm 2]. In summary, Algorithm 3 is designed to tackle composite problems of the form in (46), making the assumptions that h is L-smooth and g is a proper lower-semicontinuous function such that F≜h+g is bounded from below and coercive. This means that ${lim}_{∥x∥\to \infty }\;F(x)=+\infty$. It’s important to note that neither h nor g are required to be convex. Algorithm 3 can be summarized as follows:

**Algorithm 3:** Accelerated PG algorithm

1:	Initialize: μ, s=1, q=2, l, $x^{0}$, $x^{1}$, k=1.
2:	**repeat**
3:	$y^{k}=x^{k}+\frac{k-1}{k+2}\left(x^{k}-x^{k-1}\right)$
4:	$\Delta^{k}={max}_{t=max\{1,k-l\},\ldots ,k}\;F\left(x^{t}\right)$
5:	**if** $F\left(y^{k}\right)\leq \Delta^{k}$ **then**:
6:	$v^{k}=y^{k}$
7:	**else**:
8:	$v^{k}=x^{k}$
9:	${\bar{x}}^{k}=v^{k}-\frac{1}{L}\nabla f\left(v^{k}\right)$
10:	**for** i∈[n] **do**:
11:	solve $a^{2q}-{\overset{‾}{x}}_{i}a^{q}+\frac{2s}{q\mu L}a^{s}=0$
12:	$x_{i}^{k+1}=a^{{*}^{q}}$
13:	k=k+1
14:	**until** convergence

- An extrapolation $y_{k}$ is generated as introduced in [42] for the APG algorithm (step 3).
- Steps 4 through 9 encompass a mechanism for a non-monotone update of the objective function. Specifically, $F\left(y_{k}\right)$ undergoes scrutiny concerning its relation to the maximum among the most recent l objective values. Step 9 is responsible for adjusting the gradient step accordingly. This adjustment occasionally allows $y^{k}$ to increase the objective, resulting in a situation where $F\left(y^{k}\right)$ becomes lower than the maximum objective value observed in the latest l iterations.
- Steps 11 and 12 represent the solution of the PG step using the non-convex projection method.

In the next part, we show that Algorithm 3 converges to a critical point and it exhibits a convergence rate of $O\left(\frac{1}{K}\right)$, where K is the iteration budget that is given to the algorithm.

## Definition 1 ([43])

*The Frechet sub-differential of* F *at* x *is*

(57) $$\overset{ˆ}{\partial }F(x)≜\left\{u\;:\underset{y\neq x}{lim}\;\underset{y\to x}{lim}\;\frac{F(y)-F(x)-u^{⊤}(y-x)}{∥y-x∥}\geq 0\right\}.$$

*The sub-differential of* F *at* x *is*

(58) $$\partial F\left(x\right)≜\{u\;:\exists x^{k}\to x,F\left(x^{k}\right)\to F\left(x\right)\;\text{and}\;u^{k}\in \overset{ˆ}{\partial }F\left(x^{k}\right)\to u\;\text{as}\;k\to \infty \}.$$

## Definition 2

([43]) x *is a critical point of* F *if* 0∈∂g(x)+∇h(x).

By comparing (46) and (45), it can be realized that the functions g(x) and h(x) in definition 2 are equal to $∥x{∥}_{p}^{p}$ and $\frac{\mu }{2}f(x)$ respectively.

## Theorem 2

*The sequence* $x^{k}$ *generated from* Algorithm 3 *has at least one limit point and all the generated limit points are critical points of* (45). *Moreover, the algorithm converges with rate* $O\left(\frac{1}{K}\right)$, *where* K *is the iteration budget given to the algorithm.*

### Proof

It can easily be verified that our problem in (45) satisfies all required assumptions for Algorithm 3. Indeed,

1. The function $g(x)=∥x{∥}_{p}^{p}$ is a proper and lower semi-continuous function.
2. The gradient of $h(x)=\frac{\mu }{2}f(x)$ is $\overset{‾}{L}$-Lipschitz smooth, i.e., $∥\nabla h(x)-\nabla h(y)∥\leq \overset{‾}{L}∥x-y∥$ for all $x,y\in R^{n}$, with $\overset{‾}{L}=\frac{\mu }{2}L$.
3. F(x)=g(x)+h(x) is bounded from below, i.e., F(x)≥0.
4. ${lim}_{∥x∥\to \infty }\;F(x)=\infty$.
5. Let $𝒢(x)≜x-{prox}_{g/\lambda }(x-\nabla h(x)/L)$. From [43, 44], $∥𝒢(x){∥}^{2}$ can be used to measure how far x is from optimality. Specifically, x is a critical point of (46) if and only if 𝒢(x)=0.
6. The introduced non-convex projection method is an exact solution for the proximal gradient step. This is because it is based on finding the roots of a polynomial of order 2q in (56).

Therefore, from Theorem 4.1 and Proposition 4.3 of [41], the sequence generated by Algorithm 3 converges to a critical point of (45). Additionally, ${∥𝒢\left(x^{k}\right)∥}^{2}$ converges with rate $O\left(\frac{1}{K}\right)$, thereby completing the proof.

## Remark 1

*The global convergence of several exact iterative methods that solve* (46) *has been explored, under the framework of Kurdyka–Lojasiewicz (KL) theory, in various additional literature including* [45, 43, 46, 47, 48]. *Other work (see* [49] *and references therein) considered the linear convergence of non-exact algorithms with relaxations on the assumptions of* KL *theory, however, it is difficult to verify that the sequence generated by* Algorithm 3 *satisfies the relaxed assumptions stated in* [49].

## 6 Numerical Results

In this section, we present numerical examples to illustrate the application of the $\ell_{p}$ quasi-norm ADMM (pQN-ADMM) algorithm, as expounded in Algorithm 1, and the non-convex projection method delineated in Algorithm 2. Within each of the ensuing examples, we conduct comparative analyses with the convex $\ell_{1}$ relaxation solution, achieved through the use of the MOSEK solver [50], and alternative $\ell_{0.5}$-based solutions previously proposed in the literature.

The degree of the polynomial for which the roots are determined during the non-convex projection step depends on the value of q in the context of $p=\frac{s}{q}$. It might lead one to speculate that the computational complexity of the non-convex projection step is contingent on the specific value of p, suggesting that lower values of p result in slower algorithm performance. In order to explore this aspect, we systematically performed the non-convex projection step 200 times on a vector of 1024 elements, as part of a sparse vector reconstruction example. Throughout this process, we systematically varied the values of the parameter p, considering a range of p values, specifically $p\in \left\{\frac{1}{2},\frac{1}{3},\frac{1}{4},\frac{1}{5}\right\}$. The average time to perform the non-convex projection for the entire vector, where the roots of (26) for each p are computed using the ”root” command in MATLAB, is observed to be nearly constant, approximately 0.03 seconds. Furthermore, our numerical experiments in this particular example indicated that for $p\in \left\{\frac{1}{3},\frac{1}{4},\frac{1}{5}\right\}$, no substantial improvement over the $\ell_{0.5}$ case was observed. As a result, these cases are currently undergoing further investigation and are not included in the numerical results section.

### 6.1 Sparse Vector Recovery (SVR)

In this section, we implement a sparse vector reconstruction problem and compare the solution of the pQN-ADMM algorithm with the $\ell_{1}$ relaxation along with $\ell_{0.5}$-FL and LAIT, as presented in [51] and [52], respectively.

Let $n=2^{10}$ and m=n/4, randomly construct the sparse binary matrix, $M\in R^{m\times \frac{n}{2}}$, with a few number of ones in each column. The number of ones in each column of M is generated independently and randomly in the range of integers between 10 and 20, and their locations are randomly chosen independently for each column. Let U=[M,−M], which is the vertical concatenation of the matrix M and its negative. Following the same setup in [53], the column orthogonality in U is not satisfied. Let $x_{opt}\in R^{n}$ be a reference signal with ${∥x_{opt}∥}_{0}=\lceil 0.2n\rceil$, where the non-zero locations are chosen uniformly at random with the values following a zero mean, unit variance Gaussian distribution. Let $v={Ux}_{opt}+n$ be the allowable measurement, where $n\in R^{m}$ is a Gaussian random vector with zero mean and co-variance matrix $\sigma^{2}I_{m\times m}$, where I is the identity matrix. The sparse vector is reconstructed from v by solving (5) with 𝒱={x:∥Ux−v∥/∥v∥−ϵ≤0}, where $\epsilon =\frac{3\sigma }{∥v∥}$. All the algorithms are terminated if $∥x^{k}-x^{k-1}∥/∥x^{k-1}∥\leq 10^{-4}$ or a budget of 200 iterations is consumed.

**Figure 1:**
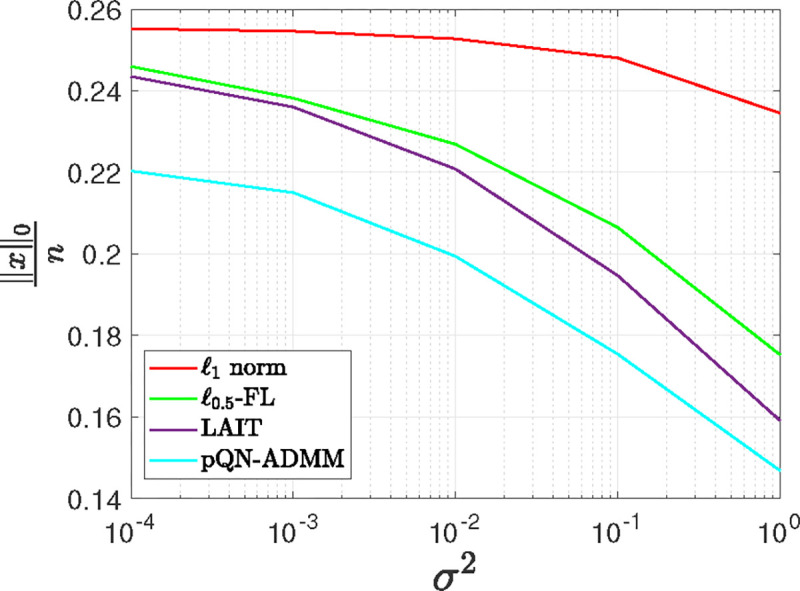
The influence of noise variance on the sparsity of solutions generated by the pQN-ADMM, $\ell_{0.5}$-FL, LAIT algorithms, and $\ell_{1}$ norm minimization.

Figure 1 depicts the correlation between sparsity levels and noise variances concerning solutions derived through $\ell_{1}$ norm minimization, $\ell_{0.5}$-FL, LAIT, and pQN-ADMM techniques. A threshold of 10^−6^ was imposed, designating entries of the solution vector as zero if they fell below this threshold. The reported outcomes are based on the average results obtained from 20 independently conducted random iterations. Notably, it becomes evident that the pQN-ADMM algorithm consistently yields solutions with higher sparsity levels in comparison to its counterpart baseline methods, across a range of $\sigma^{2}$ values. As $\sigma^{2}$ increases, the sparsity level for all approaches decreases, attributable to the heightened scarcity of information pertaining to the original signal within the realization vector, thereby compromising the precision of the reconstruction process.

### 6.2 Rank Minimization Problem (RMP)

Within this section, our primary focus is directed towards the exploration of the pQN-ADMM algorithm within the RMP framework, as presented in Section 4. We commence by engaging in a matrix completion scenario, presenting an extensive comparative analysis pitting the pQN-ADMM algorithm against various baseline methods rooted in the Schatten- p quasi-norm framework.

Additionally, we delve into a time domain system identification example. Notably, we restrict our comparative analysis to the convex nuclear norm. This singularity in focus arises from the unique constraint nature of the problem at hand, specifically the Hankel constraint. To the best of our knowledge, there are no other Schatten-p-based algorithms capable of addressing constraints of this specific nature in the proposed formulation. This serves to underscore the remarkable versatility of the pQN-ADMM algorithm in handling a broad spectrum of constraints, be they within the vector or matrix domain.

#### 6.2.1 Matrix Completion

In this section, we apply our algorithm (pQN-ADMM) to a matrix completion example and compare the result to the matrix iterative re-weighted least squares (MatrixIRLS) [54, 55], truncated iterative re-weighted unconstrained Lq (tIRucLq) [56] and iterative re-weighted least squares (sIRLS-p & IRLS-p) [57] algorithms. The matrix completion problem is a special case of the low-rank minimization where a linear transform takes a few random entries of an ambiguous matrix $X\in R^{m\times n}$. Given only these entries, the goal is to approximate X and find the missing ones. The matrix completion problem with low-rank recovery can be approximated by,

(59) $$\underset{X}{min}\;∥X{∥}_{p,*}^{p},\;\text{s.t.}\;∥𝒜(X)-b∥\leq \epsilon ,$$

where $𝒜\;:R^{m\times n}\to R^{q}$ is a linear map with q≪mn and $b\in R^{q}$. To facilitate the application of the aforementioned algorithms, the linear transform 𝒜(X) will be reformulated as Avec(X), where $A\in R^{q\times mn}$ and $vec(X)\in R^{mn}$ represents a vector obtained by stacking the columns of the matrix X.

A random matrix $M\in R^{m\times n}$ with rank r is created using the following method: 1) $M=M_{L}M_{R}^{⊤}$, where $M_{L}\in R^{m\times r}$ and $M_{R}\in R^{n\times r}$. 2) The entries of both $M_{L}$ and $M_{R}$ are i.i.d Gaussian random variables with zero mean and unit variance. Let $\overset{ˆ}{M}=M+Z$, where $Z\in R^{m\times n}$ is a Gaussian noise with each entry being an i.i.d Gaussian random variable with zero mean and variance $\sigma^{2}$. The vector b is then created by selecting random q elements from $vec(\overset{ˆ}{M})$. Since $b=Avec(\overset{ˆ}{M})$, one can easily construct the matrix A which is a sparse matrix where each row is composed of a value 1 at the index of the corresponding selected entry in the vector b while the rest are zeros. We set m=n=100, r=5 and p=0.5. Let $d_{r}=r(m+n-r)$ denotes the dimension of the set of rank r matrices and define $s=\frac{q}{mn}$ as the sampling ratio. We assume that s=0.195 which yields to q=1950. It can be realized that $\frac{d_{r}}{q}<1$. We set =0.1, $\epsilon =10^{-3}$, and let the algorithms terminate if a budget of 1000 iterations is reached. In order to compare the results from different algorithms, we consider the average of 50 runs for two measures: a) the Relative Frobenius Distance (RFD) to the matrix M, b) the Relative Error to Singular (REtS) values of M.

**Figure 2:**
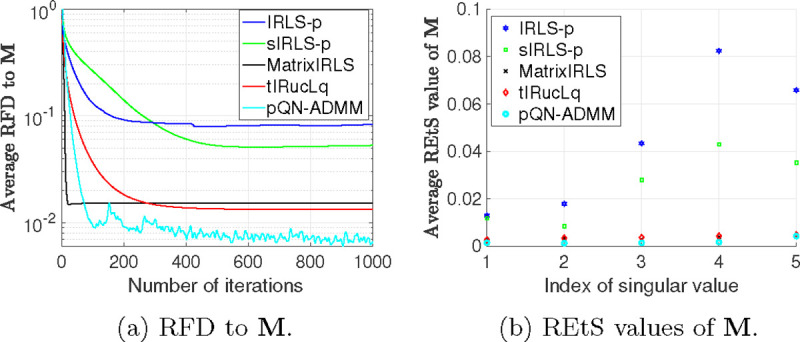
The RFD and REtS average values.

In Figures 2a and 2b, we report the average RFD and REtS values for all the algorithms. Despite that, all the baselines are designed to exploit the specific structure of the matrix completion problem, described in (59), while the proposed pQN-ADMM doesn’t, it is competitive against them all. This in turn shows the effectiveness of the pQN-ADMM algorithm in solving the rank minimization problems without requiring any prior information about the structure of the associated convex set.

#### 6.2.2 Time domain system identification

We consider a stable Single Input Single Output (SISO) system operating in discrete time, wherein the input vector $u\in R^{T}$ corresponds to a temporal span denoted by T, representing the number of input samples. The system is characterized by an impulse response consisting of a fixed number of samples denoted as n. The resultant output of the system is represented by $y\in R^{m}$. However, in practical scenarios, only noisy realizations, denoted as $\overset{ˆ}{y}$, are observable. This realization is expressed as $\overset{ˆ}{y}≜y+z=h⊛u+z$, where $h\in R^{n}$ signifies the system’s original impulse response, $z\in R^{m}$ is a random vector with entries drawn independently from a uniform distribution within the range [−0.25, 0.25], and ⊛ denotes the convolution operator.

Exploiting the window property of convolution, which asserts that m=n+T−1, we establish the relationship among the components $u_{i}$, $h_{i}$, and $y_{i}$ through the linear convolution relation $y_{i}=\sum_{j=-\infty }^{\infty }\;h_{j}u_{i-j}$. Herein, $u_{i}$, $h_{i}$, and $y_{i}$ represent the i th components of the vectors u, h, and y, respectively. To succinctly represent the convolution, let $T\in R^{m\times n}$ be the Toeplitz matrix formed by the input u, allowing us to express $h⊛u={hT}^{⊤}$. Furthermore, assuming $x\in R^{n}$ to be an impulse response variable, we introduce $X\in R^{n\times n}$ as a Hankel matrix formed by the entries of x. From [58, 59, 60, 61], the minimum order time domain system identification problem can be formulated as:

(60a) $$\underset{X}{min}\;Rank(X),$$

(60b) s.t.X=Hankel(x),

(60c) $${∥\overset{ˆ}{y}-xT^{⊤}∥}^{2}\leq \epsilon ,$$

(60b) ensures that X is a Hankel matrix and (60c) holds to make the result by applying the input, u, to the optimal impulse response, x, fit the available noisy data, $\overset{ˆ}{y}$, in a non-trivial sense. Defining the convex set $𝒞≜\{X\in R^{n\times n}:{∥\overset{ˆ}{y}-xT^{⊤}∥}^{2}-\epsilon \leq 0,X=Hankel(x)\}$, (60) can be cast as:

(61) $$\underset{X\in 𝒞}{min}\;\;Rank(X),$$

which is clearly identical to the problem in (1). The problem was solved using the same pQN-ADMM approach discussed in Section 4.

Let T=m=50 and n=40. It is pertinent to note that m<T+n−1, is a reasonable assumption aligning with practical applications where only a specific window is available to observe the output. The simulation is conducted across 5 distinct original system orders denoted by η∈{2,4,6,8,10}. An input vector, u, is generated, with its elements being independent and following a uniform distribution over the interval [−5, 5]. For each η:

1. Fifty random stable systems are generated using the ‘drss’ command in MATLAB.
2. The generated input is applied to each system, yielding the corresponding noisy output $\overset{ˆ}{y}$.
3. Given the output $\overset{ˆ}{y}$, the problem specified in (60) is solved, and the rank of the corresponding system is computed using singular value decomposition.
4. The obtained results are averaged to derive the corresponding average rank for each original η.

**Figure 3:**
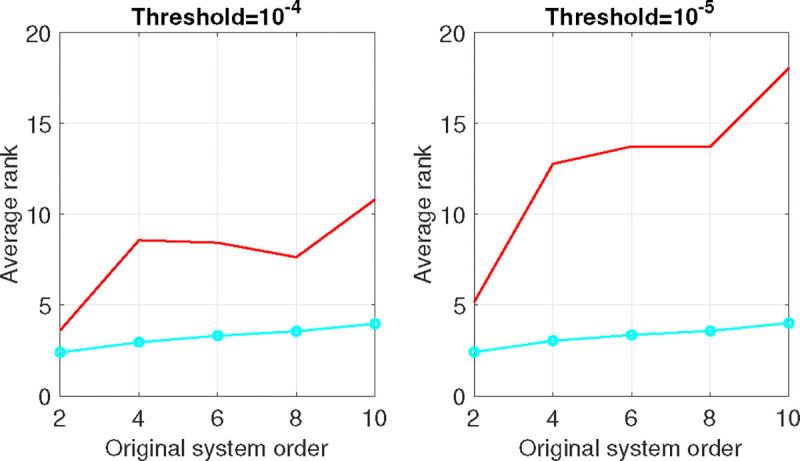
Average rank vs. original system order. Red and cyan colors are for the nuclear norm and pQN-ADMM algorithm respectively.

Figure 3 presents the average rank results obtained through the nuclear norm and pQN-ADMM heuristics. The outcomes correspond to two distinct threshold values, wherein the threshold is defined as the value below which the singular value is considered zero. Notably, the introduced pQN-ADMM approach demonstrates superior performance compared to the nuclear norm heuristic for both threshold values. Furthermore, as the threshold value decreases from 10^−4^ to 10^−5^, the pQN-ADMM’s behavior remains consistent, while the average rank for the nuclear norm exhibits an increase. This observation underscores the robustness of the derived pQN-ADMM relative to the nuclear norm approach.

Table 1 provides the standard deviation values for the algorithms. It is evident that the standard deviation remains constant for the pQN-ADMM when altering the threshold; conversely, it increases for the nuclear norm as the threshold value decreases.

### 6.3 Accelerated Proximal Gradient (APG) Algorithm

In this section, we present numerical results for the APG method, as outlined in Algorithm 3. Our primary objective is to address the minimization problem (45) with $f(x)=∥Ax-b{∥}^{2}$.

Consistent with the approach in [62], we initiate the process by generating the target signal $x^{*}$ through:

(62) $$x_{i}^{*}=\left\{\begin{array}{ll} \Theta_{i}^{\left(1\right)}10^{3\Theta_{i}^{\left(2\right)}}, & \forall \;i\in \Lambda ,\\ 0, & \forall \;i\in \left[n\right]\backslash \Lambda ; \end{array}\right.$$

where the design parameters Λ⊂[n], and $\Theta_{i}^{\left(1\right)}$, $\Theta_{i}^{\left(2\right)}$ for i∈Λ are chosen as follows:

1. The index set Λ⊂[n] is constructed by selecting a subset of [n] with cardinality s uniformly at random;
2. ${\left\{\Theta_{i}^{\left(1\right)}\right\}}_{i\in \Lambda }$ are Independent and Identically Distributed (IID) Bernoulli random variables taking values \pm 1 with equal probability;
3. ${\left\{\Theta_{i}^{\left(2\right)}\right\}}_{i\in \Lambda }$ are IID uniform [0, 1] random variables.

The measurement matrix $A\in R^{m\times n}$ is constructed as a partial Discrete Cosine Transform (DCT) matrix, with its rows corresponding to m<n frequencies. Specifically, these m indices are selected uniformly at random from the set [n]. The noisy measurement vector $b\in R^{m}$ is subsequently defined as $b=A\left(x^{*}+\epsilon_{1}\right)+\epsilon_{2}$, where $\epsilon_{1}$ and $\epsilon_{2}$ are IID random vectors with entries following zero mean Gaussian distributions with variances $\sigma_{1}^{2}$ and $\sigma_{2}^{2}$ respectively.

In our experiments, n=4096, s=⌈0.5m⌉ and the APG algorithm memory to 5, i.e., l=5 in Algorithm 3. Following the medium noise setup in [63], we set $\sigma_{1}=0.005$, $\sigma_{2}=0.001$.

For the objective function $f(x)=∥Ax-b{∥}^{2}$, the Lipschitz constant is given by $L=2∥A{∥}^{2}$. Our experimental design encompasses varying values of m, representing the number of noisy measurements, and μ, serving as the trade-off parameter in (45). For each unique combination of (m,μ), we conduct 20 random instances of the triplet $\left(x^{*},A,b\right)$ to account for the inherent statistical variability of the problem. Each random instance is subsequently solved using Algorithm 3, and the average performance is reported. The termination criterion for Algorithm 3 is defined as the relative error between consecutive iterates satisfies $∥x^{k}-x^{k-1}∥/∥x^{k-1}∥\leq 10^{-5}$. In our experiments, we conducted a comparative analysis of solving (45) for p=0.5 against p=1, corresponding to $\ell_{1}$-optimization for sparse recovery. Specifically, for p=0.5, denoting $\ell_{0.5}$ minimization, we employed Algorithm 3, referred to as $\ell_{0.5}$ exact. Additionally, we utilized Algorithm 2 from [64], denoted as $\ell_{0.5}$ approx. Conversely, for p=1, where the $\ell_{1}$-minimization problem is convex, we employed the FISTA algorithm from [65]. The solutions are denoted as $\bar{x}$, while the target signal, derived from (62), is denoted as $x^{*}$. In Algorithm 3, we initialized $x^{0}$ as a zero vector, and $x^{1}$ was set to the $\ell_{1}$ norm solution.

**Figure 4:**
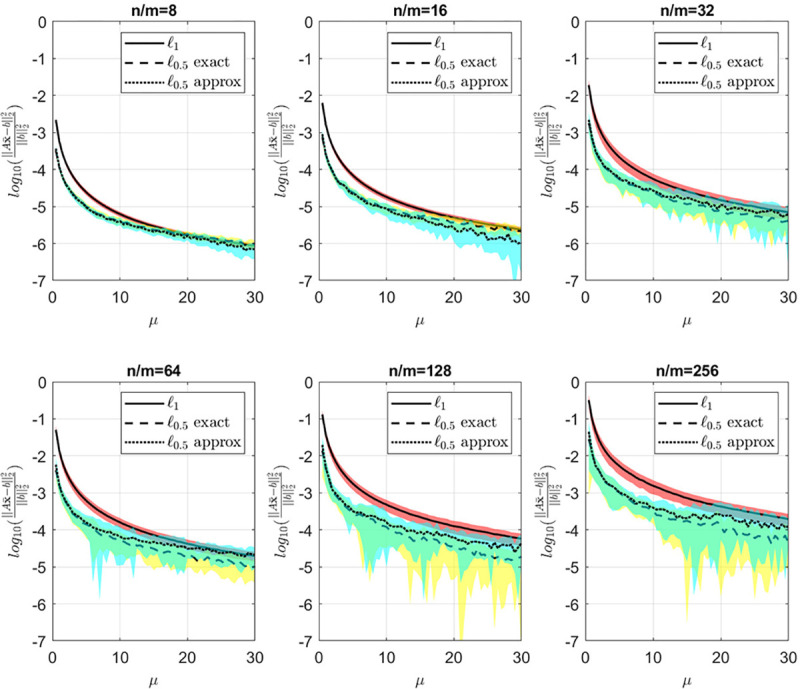
Average error vs μ for different values of n/m. Yellow and cyan shades are the standard deviations for the exact and approximate $\ell_{0.5}$ quasi-norms respectively.

**Figure 5:**
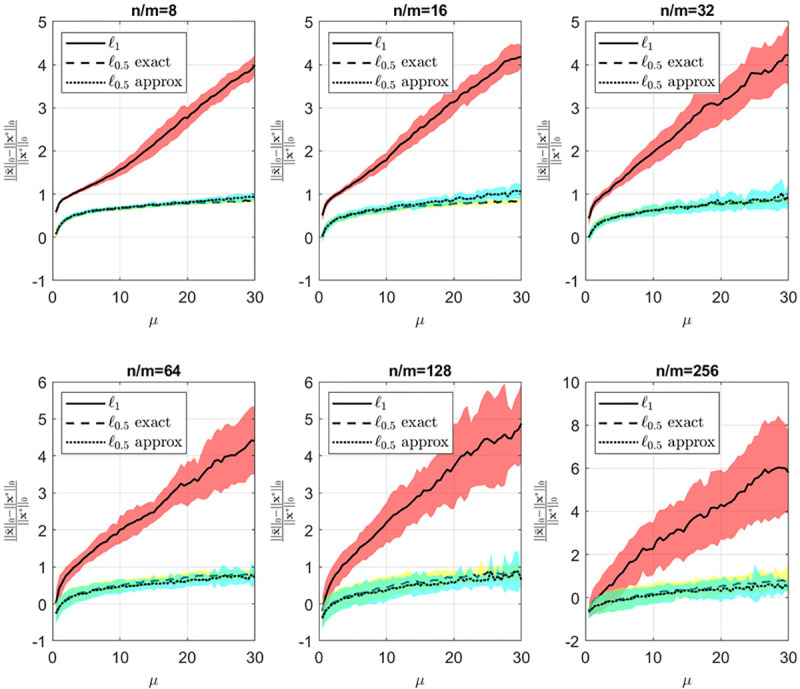
Sparsity vs μ for different values of n/m. Yellow and cyan shades are the standard deviations for the exact and approximate $\ell_{0.5}$ quasi-norms respectively.

Figures 4 and 5 illustrate the relationship between average error, sparsity, and μ for various values of n/m. A discernible trend is observed wherein the average error decreases while sparsity increases with an increase in μ. When μ is small, greater emphasis is placed on the loss function, emphasizing $\ell_{0.5}$ quasi-norm minimization. Consequently, the sparsity level, as depicted in Figure 5, remains low. Conversely, for higher values of μ, more weight is assigned to the regularization term’s minimization, resolving $∥Ax-b{∥}^{2}$, resulting in decreased error (Figure 4) accompanied by an increase in sparsity.

**Figure 6:**
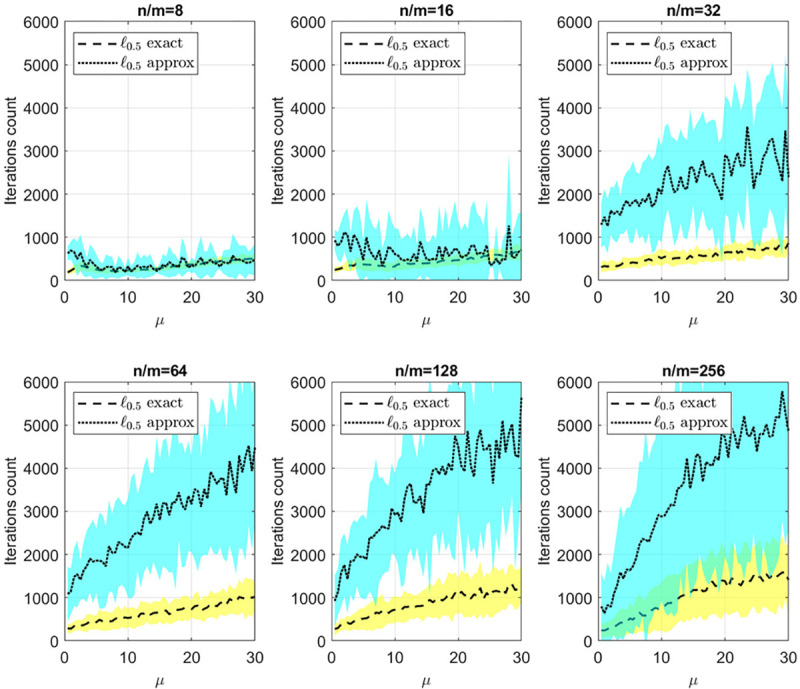
Iterations count vs μ for different values of n/m.

Figure 6 provides insight into the statistics of the number of iterations until convergence for both the $\ell_{0.5}$ exact and approximate algorithms. Notably, with a sufficient number of available realizations, specifically for n/m=8 and n/m=16, both algorithms require approximately the same number of iterations. However, as the number of available realizations decreases, particularly for n/m=32 and higher, the exact proximal solution exhibits a significantly lower number of iterations to converge. This observation, coupled with the findings in Figures 4 and 5, suggests that our algorithm not only yields a comparable solution to the approximate method but also converges with fewer iterations.

## 7 Conclusion

In this paper, we introduced a non-convex ADMM algorithm, denoted as pQN-ADMM, designed for solving the $\ell_{p}$ quasi-norm minimization problem. Significantly, our proposed algorithm serves as a versatile approach for tackling $\ell_{p}$ problems, as it does not rely on specific structural assumptions for the convex constraint set. Moreover, we delved into the problem of solving a non-convex relaxation of RMPs utilizing the Schatten- p quasi-norm. This relaxation was established as the $\ell_{p}$ minimization of the singular values of the variable matrix, rendering it amenable to the pQN-ADMM algorithm. For scenarios involving constraints defined by differentiable functions with Lipschitz continuous gradients, a proximal gradient step was employed, mitigating the need for a convex projection step. This enhancement not only accelerates the algorithm but also ensures its convergence. Illustrating the numerical results, we applied the pQN-ADMM to diverse examples, encompassing sparse vector reconstruction, matrix completion, and system identification. The algorithm demonstrated competitiveness against various $\ell_{p}$-based baselines, underscoring its efficacy across a spectrum of applications.

## Figures and Tables

**Table 1:** Standard deviation for different threshold values.

	Threshold = 10^−4^			Threshold = 10^−5^		
	*η*=2	*η*=6	*η*=10	*η*=2	*η*=6	*η*=10
**Nuclear norm**	2.3907	6.6668	7.2572	6.9877	11.2638	11.7854
**pQN-ADMM**	0.5292	0.9042	1.0861	0.5325	0.9113	1.0861

## Data Availability

Not applicable
